# Pregnancy and reproductive health in women with bipolar disorder at the Arrazi Psychiatric University Hospital in Salé

**DOI:** 10.1192/j.eurpsy.2025.785

**Published:** 2025-08-26

**Authors:** N. Ait Bensaid, M. Sabir, F. El Omari

**Affiliations:** 1 arrazi psychiatric hospital, Sale, Morocco; 2 HAS, arrazi psychiatric hospital, Sale, Morocco

## Abstract

**Introduction:**

Bipolar disorder, like other severe mental illnesses, is considered to be a condition with an increased risk of unsafe sexual practices [1]. Unsafe sex in bipolar disorder has been empirically linked to manic episodes due to symptoms of hypersexuality, cognitive impairment and substance and alcohol abuse.

Unprotected sex poses health risks for both sexes in terms of sexually transmitted diseases, but for women it also means a risk of unplanned pregnancy. Unplanned pregnancies can have negative consequences for the health, social and psychological lives of women and children [2].

Translated with DeepL.com (free version)

**Objectives:**

To assess reproductive health and unplanned pregnancies in patients with bipolar disorder followed and hospitalised at the Arrazi psychiatric hospital in Salé.

**Methods:**

This was a descriptive cross-sectional study using a questionnaire including sociodemographic and clinical criteria as well as questions on the sex life of patients with bipolar disorder type I or II, questions on contraception, and on pregnancies and their outcomes.

Inclusion criteria: women with bipolar disorder type I or II.

Exclusion criteria: psychosis, intellectual disability.

**Results:**

The average age of the participants was 33 years. 68% were married and 57% had children. The majority were unemployed (87%). 85% had a substance use disorder. 61% had type I bipolar disorder and 44% were hospitalised, with the remainder receiving outpatient treatment. Almost all the patients were sexually active at the time of the study, and 88% had only one sexual partner. 77% were using contraception, mainly the pill. The average age of first pregnancy was 22 years. 66% of pregnancies were unplanned. 89% gave birth and 11% had abortions.

**Conclusions:**

Bipolar disorder, like other serious mental illnesses, is considered to be a condition with an increased risk of unsafe sexual practices. Female patients with bipolar disorder have an early age of onset of sexual activity. This leads to frequent unplanned pregnancies. These unplanned pregnancies can have negative consequences for the health, social and psychological life of women and children. Clinicians must be aware of reproductive health and take steps to improve access to family planning when treating young women with bipolar disorder, in order to avoid negative consequences.

**Disclosure of Interest:**

None Declared

